# Distributed Watchdogs Based on Blockchain for Securing Industrial Internet of Things †

**DOI:** 10.3390/s21134393

**Published:** 2021-06-26

**Authors:** JongHyup Lee, Taekyoung Kwon

**Affiliations:** 1 Department of Financial Mathematics, Gachon University, Seongnam 13120, Korea; jonghyup@gachon.ac.kr; 2 Graduate School of Information, Yonsei University, Seoul 03722, Korea

**Keywords:** IIoT, software update, blockchain

## Abstract

The Industrial Internet of Things (IIoT) could enhance automation and analytics in industrial environments. Despite the promising benefits of IIoT, securely managing software updates is a challenging problem for those critical applications. This is due to at least the intrinsic lack of software protection mechanisms in legacy industrial systems. In this paper, to address the challenges in building a secure software supply chain for industrial environments, we propose a new approach that leverages distributed watchdogs with blockchain systems in protecting software supply chains. For this purpose, we bind every entity with a unique identity in the blockchain and employ the blockchain as a delegated authenticator by mapping every reporting action to a non-fungible token transfer. Moreover, we present a detailed specification to clearly define the behavior of systems and to apply model checking.

## 1. Introduction

The Industrial Internet of Things (IIoT) has emerged as a new technology that adopts the Internet of Things (IoT) for industrial applications. IIoT has a similar configuration to the IoT. Numerous IoT devices continuously monitor industrial areas and regularly report the sensing data to a sink node, which is designated to collect data. Industrial automation and analytics could benefit from using the collected data in IIoT [1]. Thus, many companies are actively working on building the new IoT ecosystems [2].

One of the traditional concerns for industrial devices has been an operational failure, referred to as a crash. To deal with this problem, a *watchdog* timer circuit was developed to check whether the devices are working properly or not, by regularly sending a query signal and monitoring the output signals [3,4]. However, the crash of industrial devices is getting more complicated because smart industrial devices have become reprogrammable via software updates. This tendency has led to the evolution of the crash failure model to the *Byzantine failure* model, wherein industrial devices may be compromised and made to behave maliciously by remote attackers.

The software updates are delivered through the software supply chain. Recently, Over-The-Air (OTA) software updates based on wireless communications have been widely adopted in IIoT environments [5] consisting of a number of small devices, such as wireless sensors. The OTA update is an appropriate way to manage such a large number of devices. Large and scalable IIoTs are usually based on mesh or ad hoc networks, and their external connections are restricted only within a time window [6].

Unfortunately, securing OTA software updates in IIoT environments is a challenging problem. The security concerns of the software update are recurrently addressed in a variety of literature [7,8,9]. NIST published a technical paper on secure software updates [10] and analyzed the incidents and attack models of software supply chains. The attackers could hijack software updates and abuse the code signing. Attackers could resend old software updates or make them be delivered to the wrong targets. In the latter cases, although the update package is still legitimate, the targets could be put in danger. In 2017, mismatched software updates were installed on hundreds of IoT smart-lock devices, which caused the failure of some automatic check-in services of Airbnb [11]. The illegitimate software updates can harm the system itself. San et al. [12] showed that the attackers could stealthily inject instructions into Field Programmable Gate Array (FPGA) firmware to intrude hardware systems.

Software updates should be protected by carefully dealing with the software supply chain in IIoT systems. Current protection mechanisms using end-to-end means, such as code signing [13,14], have been rigorously tested, but basic protection mechanisms are still insufficient in the industrial environments [15]. Industrial devices should also be protected from internal attacks [15] launched by compromised nodes that are only discoverable by Internet scanning or pre-scanned databases (e.g., Shodan) [16].

**Watchdog.** Advanced watchdog approaches [17,18,19,20] in the Byzantine failure model are well known Intrusion Detection Systems (IDS). A watchdog observes the behavior of the target devices. Once a watchdog observes that one of a number of predefined behavior patterns is happening, it reports that event to a centralized entity or shares it with others in a distributed manner for further actions. The watchdog approach has been widely adapted. A host-based watchdog monitors a local system, and a network-based watchdog analyzes network traffic [21,22]. In particular, the distributed and network-based watchdog approaches are suitable for monitoring a number of devices in the new IIoT environment.

In the distributed watchdog system, all processes heavily depend on the majority voting of the participating nodes. However, authenticating reports and votes has been a long-standing and hard challenge [23] in distributed watching models. To solve this problem, blockchain-based approaches for Collaborative Intrusion Detection Systems (CIDS) have recently been proposed in [23,24]. The blockchain [25] enables distributed, immutable time-series records of validated transactions. Hence, it can be a solution for sharing authenticated status/results for distributed watchdogs. However, most consensus algorithms require a high level of computation or communicational overhead. The blockchain approach must be carefully applied to power constrained IIoT devices.

**Our Approach.** To summarize, in building a secure supply chain of software updates, we address the following problems: (1) we need a method to check the validity of the software updates in both text (binary object) and context (trace and metadata); and (2) we have to efficiently handle the data sharing and trust management of the distributed watchdogs. To address these problems, we propose a new distributed watchdog method, called “IndWatch”. We leverage a blockchain and protect every entity and every action by converting them into controllable, non-fungible entities in the blockchain system. We assign an account (or an address) to all entities, from tangible entities (e.g., watchdogs) to intangible entities (e.g., the binary objects). In addition, the actions of all entities are mapped by transferring an event token from the initiator’s account to the target’s address. For example, when a watchdog monitors its coverage area and detects one of the predefined events on a binary object, it sends an event token to the address of the observed binary object. The balance of the event tokens represents the reputation of the entity. When the balance falls below a threshold, the entity (i.e., binary objects or relaying nodes) is isolated from the software supply chain. Every transaction of the token transfer is checked by the underlying blockchain system; thus, the originator is authenticated (a transaction requires the owner’s valid digital signature) and authorized (one can transfer owned event tokens only) simultaneously.

The tangible entities of IndWatch are providers, watchdogs, and recipients. A provider prepares a binary object for the software update. A binary object is also an independent entity in IndWatch. The provider creates an account in the blockchain system for the binary object and securely binds the binary object to the account. The account can hold the context data on the binary object. The context has an invariant part (e.g., binary object fingerprints) and a mutable part (e.g., delivery traces). A watchdog monitors all incoming and outgoing binary objects and reports an event by sending an event token with the observed information to the address of the binary objects. When the binary object finally reaches the recipient, the latter validates it with the context and notifies of the arrival through the event token. The authenticated information from the event token updates the balance of the targets. Based on this balance, an agent called “judge” in the blockchain system detects corrupt binary objects or compromised relaying nodes. The judge agent is also in charge of re-issuing event tokens. When a watchdog has spent all its tokens, it requests new tokens from the judge. The judge then evaluates the watchdog and decides how many tokens it will issue for it. In this way, the reports of the watchdogs are accountable and authenticated, and the judge can control the distributed watchdogs by adjusting the reissuing rate of the event tokens.

**Contribution.** We make the following contributions with this paper:

1. We address the importance of context in the software supply chain. We emphasize the fundamental requirements that supply chain of binary objects should be followed as the provider originally intended. That is, the delivered binary objects must be able to prove the legitimacy of their delivery history as well as the integrity of their contents.
2. We leverage the blockchain as a faithful, delegated authenticator for the distributed watchdog system. The blockchain system automatically authenticates every report and authorizes the watchdogs. To do so, we map every action onto a non-fungible event token in the blockchain.
3. We identify every instance of a binary object in the supply chain as a distinguishable, traceable product. For this purpose, we propose an identity binding between a binary object and a blockchain entity. Thus, a binary object is uniquely identified with its own context and records. This is helpful to rapidly discover the product-wise problem and spot corruption.

## 2. Background and Related Work

### 2.1. Collaborative Intrusion Detection Systems

The basic model for distributed watchdogs is the Collaborative Intrusion Detection System (CIDS). In CIDS, the information of the network is gathered by distributed monitors or watchdogs. Each monitor logs the information and generates an alert when observing any misbehavior. Note that the misbehavior can be defined by a signature or a profile. Ioannis et al. proposed a distributed IDS for WSNs [18]. A watchdog monitors nearby nodes and locally detects malicious actions, to broadcast the reports to adjacent watchdogs. In the distributed manner, watchdogs then cooperatively decide which are malicious nodes through majority voting. Chen et al. also presented a watchdog-style approach for identifying malicious or faulty sensors [19]. In the proposed work, sensor nodes actively participate to keep the network healthy. A sensor node monitors the received measurement value from the neighbor sensor nodes. If the value is out of predictable range, the sensor node reports the fault result. Majority voting is also employed to decide whether a sensor node is faulty. In [20], Cho et al. systematically described the possible problems in the trust mechanisms, which mostly have to do with the reputation scoring, and suggested improvements for the monitoring and evaluation of malicious sensors. Cervantes et al. also used the distributed watchdogs to identify the sinkhole attacks in 6LoWPAN IoT networks [26]. The watchdogs analyze the nearby nodes and use reputation scores as accumulated reports for misbehavior. The malicious or erroneous behavior of intermediate nodes is monitored by the sensor nodes in the work of Pu et al. [27]. In the proposed protocol, a sensor node also operates as a watchdog for checking forwarding behaviors.

The majority voting on which distributed watchdogs is heavily dependent also has authentication issues. In IndWatch, we use reputation scoring and employ the blockchain to automatically authenticate all changes and to secure reputation scores.

### 2.2. Blockchain

Blockchain is a decentralized ledger system that is maintained without a central authority. According to its configuration, the blockchain system is categorized into public and private blockchains. However, both blockchain systems enable the same function: the authenticated ledger. Whenever a transaction is submitted to the blockchain system, self-interested authenticators, called “miners”, check the validity of the transaction and confirm the changes as “blocks”. For this purpose, distributed consensus mechanisms, e.g., proof of work and proof of stake, are used and provide an append only, verifiable record. Owing to the security of the blockchain system, crypto-currency systems employ blockchain as their infrastructure [28,29,30]. It is also being used in many other applications, e.g., financial transactions [31], proof of validity of documents [32], and IoT [33].

Among the systems based on the blockchain infrastructure, Bitcoin [28] and Ethereum [29] are the most popular. The fundamental elements of these blockchain system are the *address* and the *transaction*. A transaction is used to transfer money from an address to another address. That is, a transaction represents the state change involving the corresponding addresses. It may contain programmable trigger conditions, which are called “smart contracts”. A smart contract is operated as a program code that can be executed by itself on top of the blockchain systems. Depending on the blockchain system, the implementation and capability of smart contracts differ. In particular, Ethereum provides more generalized smart contracts. It has an execution model for smart contracts and each smart contract resides on a blockchain, meaning that is has an independent account of its own address.

## 3. IndWatch

### 3.1. Motivations

The distributed watchdog system compensates the limited coverage area by collaborative report sharing and majority voting. However, watchdogs may also be faulty or compromised, and authenticating every watchdog in a distributed setting is still challenging. The main challenges are twofold [23]: (1) data sharing; and (2) trust management. Secure data sharing requires that all participants needs to trust each other, and, with the risk of insider attacks by compromised watchdogs and servers, maintaining trustworthy results is hard for large organization such as smart factories.

The goal of the software supply chain protection is to ensure that the binary objects of the software updates are delivered along the supply chain as per the provider’s intention. The provider’s intention can be represented as: (1) target configuration; (2) delivery trace (time and segment); and (3) the integrity of the binary object. The first two factors are formed into the *context*. Recent accidents caused by forged or mismatched software updates [11] show that secure context management is an important factor for operating an entire industrial system correctly. Thus, a software patch must be applied to the device with the correct target configuration and in the order specified. However, a conventional software update is focused on the integrity of the binary object (text) itself.

Blockchain enables a mutually agreed state among distrusting participants. Due to this feature, blockchain fits the base system for distributed watchdogs as in [24]. However, using an IIoT node as a fully functioning node (or block producing node) is impractical because of high communicational cost for peer-to-peer communications. Therefore, as an alternative approach, we use the blockchain as an automated authenticator of participants and a publicly verifiable recorder of malicious nodes.

In IndWatch, a report from watchdogs is processed as a transaction to be authenticated and verified. For this purpose, every participant has dual identity: one in the IIoT network and the other in the blockchain network (i.e., address space). Additionally, the two identities must be mutually paired to authenticate their relationship. We refer to this as “identity binding”.

We summarize the requirements for distributed watchdog systems as follows:

R1 **Identity binding:** An entity specified in the system should have a dual identity, respectively working in IIoT networks and blockchain networks. The one-to-one mapping between the two identities should be verifiable.
R2 **Authenticated reports:** The reports from evaluators, watchdogs, and receivers are authenticated in terms of the origin and the integrity.
R3 **Joint decision:** An individual watchdog monitors the behavior of nodes and solely decides on the occurrence of a local event. However, to identify malicious nodes, multiple watchdogs should collaborate with each other in the same region.
R4 **Distributed management of the software supply chain:** In covering IIoT networks, the distributed approach is effective, but each entity cannot cover the whole area alone. Thus, distributed watchdogs must verify the end-to-end integrity of binary objects and identify misbehaving nodes on the software supply chain.
R5 **Trustless watchdogs:** The watchdog is also a node that can malfunction or be compromised. Thus, the regulation is required for the evaluation process, in addition to the authenticated reports.

### 3.2. Blockchain-Based Reputation System

To satisfy the requirements mentioned in Section 3.1, we employed a blockchain as a delegated record keeper and inspector. A blockchain maintains a single global state for all the entities in distributed systems. Every action of change in the global state of a blockchain is formed as a transaction and every transaction is validated by the block producers (i.e., miners). To apply the blockchain system to IIoT, we define two name spaces, $N_{d}$ and $N_{b}$, for the IIoT network and blockchain, respectively. We define the one-to-one mapping relation between two name spaces as follows:

**Definition** **1** (Identity Binding)**.** *The function $M_{b}$ shows one-to-one mapping from $N_{d}$ to $N_{b}$, i.e., $M_{b}:N_{d}\to N_{b}$. Likewise, $M_{d}$ is a mapping from $N_{b}$ to $N_{d}$, i.e., $M_{d}:N_{b}\to N_{d}$.*

We classify entities based on the capacity of making transactions into active entities (e.g., watchdogs and receivers) and passive entities (e.g., binary objects). An active entity can prove its binding interactively. Since the blockchain transactions contain a digital signature by default, only the owner of an address can make legitimate transactions from it. Thus, by interactively challenging, such as requesting to make a transaction from the given address, the identity binding to the blockchain address is verifiable. In addition, the token system can exclude reports from unregistered watchdogs since it has control over initial token balances.

The passive entities cannot use the interactive approach, and thus we imprint the blockchain address to produce the one-to-one mapping. A binary object does not need to create a transaction from it, but its balance needs to manage. The provider creates a legitimate address derived from the hash of the binary object and imprints the address inside of the binary object. Subsequently, the fingerprint of the binary object is created from the imprinted binary object and then managed in the context.

We also define two types of tokens: RepoToken to report an event and PenToken to penalize a target for its bad behavior. A manager in a blockchain, called “judge”, mints the tokens and provides them to the *evaluators* (watchdogs and recipients). The evaluators have actionable accounts in the blockchain. In other words, they generate transactions originating from the accounts. They can transfer their own tokens to other accounts. By contrast, the binary objects and the segments have non-actionable accounts; they receive the tokens but cannot transfer them to others. We call the tokens in an actionable account “spendable”, or otherwise “unspendable”. The balance of spendable and unspendable tokens are denoted by $B_{t}^{S}\left(a\right)$ and $B_{t}^{U}\left(a\right)$, respectively, where *t* is a token type and *a* is the address. Then, we define the reputation score as follows:

**Definition** **2** (Reputation score)**.** *The reputation score of a node n, S(n), is calculated as: $S\left(n\right)=I\left(M_{b}\left(n\right)\right)-B_{\mathrm{PenToken}}^{U}\left(M_{b}\left(n\right)\right)$, where I(a) is the initial balance of the address a given during the registration. Based on the tokens, the evaluation process is defined as a transaction that transfers a token from an evaluator’s address watchdog to a target’s address.*

In this way, the actions and results of the evaluation of the watchdogs are recorded within the blockchain, where every change is validated. If the identities of $N_{d}$ and $N_{b}$ are bound (R1), then we can ensure that the reputation score based on the blockchain is verified and only authorized entities can update the reputation score (this satisfies R2). In addition, we can determine the global state of the reputation score from the blockchain system (R3). During monitoring, watchdogs collaboratively find misbehavior and inconsistencies of packets along the software supply chain. The blockchain system plays a role as an aggregator of authenticated monitoring reports from watchdogs (R4). The judge can regulate misbehaving watchdogs by adjusting the issue rate (R5), and its core part is implemented in a smart contract, whose executions are publicly verifiable, in the blockchain system.

### 3.3. Overview

**Participants and Definitions.** In IndWatch, we assume that a software update supply chain has the following participants: providers, watchdogs, segments, recipients, and judges. The software update supply chain is a route in the wireless network from a provider to a recipient. The goal of the supply chain is to deliver a binary object for updating the software of the recipient’s industrial device. The binary object is in the form of a patch or an executable. A set of sequential nodes within the coverage of a watchdog is modeled as a “segment”. Watchdogs monitor the packets in the supply chain and report the event they observed to the judge. The judge can be implemented as smart contracts and supporting modules on the blockchain systems.

**Figure 1:**
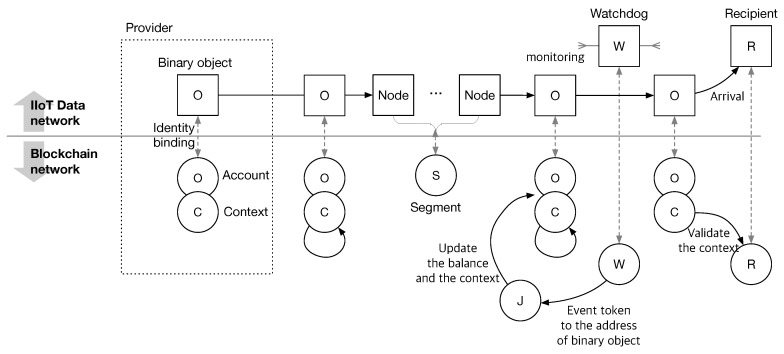
Overall process of IndWatch. Every entity has a dual identity in the blockchain and the IIoT network.

**Process.** Figure 1 shows the overall process of IndWatch. Every participant, except for the judge has a dual identity in the name space of the IIoT data network ($N_{d}$) and blockchain ($N_{b}$) and its correspondence is shown as a dashed line in Figure 1. The binary object also has its account in $N_{b}$. The account has the information on the token balance and the context of the binary object. The provider prepares a binary object and creates a blockchain address for it. The provider then sets an identity binding between the binary object and the account and sends the binary object through nodes. A watchdog monitors the delivery of the binary object at every segment. The watchdog reports the event from the observation of the binary object by sending an event token containing the observed information to the account of the binary object. While processing the token, the judge updates the balance and context of the binary object and decides whether the binary object is corrupted or not, if the balance goes zero. The judge then notifies network operators of the event. Similar to the binary object, when the balance of a segment is negative, the judge labels the segment as corrupted, and the network operator excludes the part from the supply chain.

**Balance and Reputations.** When an entity is initialized, it registers itself to the blockchain and claims the initial balance of the event tokens. For the entities of non-actionable accounts, the balance of the token represents their initial credit. The initial balance is decided by an evaluation process. For the entities of actionable accounts, the initial balance represents how many reports the entity can generate by default.

## 4. Design

In this section, we present detailed explanations on the process of IndWatch and any design considerations for the realization of IndWatch in an existing blockchain system, such as Ethereum.

### 4.1. Token System

At the core of IndWatch is a token system within the blockchain system to authenticate every event and to control the evaluators. We define two types of *event* tokens: RepoToken and PenToken. The event tokens are used to report updated data. In the blockchain, a token system consists of the balances of all the participants and the methods to securely handle the balance, such as token transfers between addresses. Thus, the token system can be implemented as a smart property (e.g., Colored coin [34] in Bitcoin) or as an independent smart contract extended from the standard token system (e.g., ERC-20 [35] or ERC-721 [36] in Ethereum).

Each entity in IndWatch has its own balance in the token system. Whenever the balance is updated, the judge in the token system investigates the change to discover corrupted binary objects or segments, a process which is described in Section 4.5.

When an evaluator reports an event, it transfers the tokens from its address to the target’s address. Miners then validate the transactions before including them into a working block. During the validation, a miner checks: (1) that the transaction sender is the valid origin of the transaction; (2) that the recipient address is valid; and (3) that the sender has sufficient tokens for the transfer. If any of these conditions is not satisfied, the transaction is rejected and the reputation of N (i.e., balance) is unchanged.

Interestingly, the tokens for watchdogs can provide the following features in IndWatch. First, every token transaction shows *who* gives *which* token to *whom*. Second, each entity has a limited quantity of tokens. Thus, we can also evaluate the evaluators. Malfunctioned evaluators are easily detected since every evaluator leaves immutable token records on the blockchain. Once all tokens are used, it can recharge the tokens; however, the number of renewed tokens depends on the judge.

### 4.2. Provider

The provider generates the binary object for deliver and creates a context for it. The context of the binary object consists of: (1) a fingerprint (hashed value) of a bound binary object; (2) a target specification; and (3) delivery traces (time and segment). The first two elements are invariant for the delivery and the last element is updated during the delivery. The context information can be stored as collocated variables in a smart contract or as distributed transactions on the address. For simplicity, we assume that the context is stored in a smart contract in this study.

For a new binary object, the provider creates an address based on the hash value of the binary object. The account is used to store the context information and identify the uniqueness of the binary object. To bind the addresses and binary objects, we let the two entities mutually hold the information of each other. More formally, the provider prepares the context (C(·)) for the binary object (O) as follows:

1. Create an address for O, *a*, based on the hash value of O.
2. Assign the address to O: $M_{b}\left(O\right)\leftarrow a$.
3. Insert address *a* into the binary code of O. To do this, the provider could use a method for code marking, such as using side-effect-free instructions described in [37,38].
4. Calculate the fingerprint (i.e., hash value) of the address-imprinted binary object.
5. Put the fingerprint into the context of the binary object, C(a).

The provider then transmits the prepared binary object through relaying nodes.

### 4.3. Watchdog and Segment

A watchdog operates in the promiscuous mode to overhear packet transmissions within the wireless communication range. We may consider the nodes by the unit of *segments*. In static networks, the supply path of binary objects may consist of fixed segments between the provider and the recipients, and so the watchdog manages the fixed segments. In dynamic networks, a segment is a set of nodes located within a watchdog’s wireless coverage.

A watchdog overhears the incoming and outgoing binary objects of all nodes in a segment and reports an event by sending an event token to the address of the binary object. The event of malicious behavior is defined as the violation of the *Reliable Forward* property, which is defined as follows:

**Definition** **3** (Reliable Forward)**.** *A binary object should be forwarded by a relaying node: (1) in a reasonable time (i.e., no intentional packet drop); (2) without any modification on the payload; and (3) to the proper next node in the route.*

The watchdogs check the three conditions of Reliable Forward. To verify the conditions, a watchdog tracks the arrival and departure of each packet at all nodes in the covered segment. When a watchdog observes a new unseen packet, it creates an entry to store the fingerprint of the payload and the arrival and departure times. The entry is updated whenever a watchdog observes a packet transmission. Watchdogs maintain timers. If any received packet is not forwarded within the predefined time, it is considered as an abnormal packet drop or delay, which means the violation of the first condition of Reliable Forward. Since the entries also have the payload fingerprints, a watchdog can verify that incoming and outgoing payloads are identical for a node. Otherwise, it is the violation of the second condition. Lastly, a watchdog checks the delivery routes of binary objects. A software supply chain consists of directional delivery channels, which are used repeatedly. Hence, a watchdog can clearly identify the immediate next nodes in a segment or immediate next segments of software update packets. The third condition of Reliable Forward is verified with the knowledge about a topology near watchdogs. In this way, routing attacks, such as loophole attacks, can be detected. Note that the surveillance of watchdogs is also effective on encrypted software payloads. The Reliable Forward conditions are still applicable without inspecting the contents of payload. The payload modification can also be checked through the stored entries in watchdogs. However, with the encrypted binary objects, the identity binding cannot be verified at watchdogs since the embedded address is visible only in plain-text binary objects. It can be verified at the final recipient where the binary object is decrypted.

When observing the violation of *Reliable Forward*, a watchdog transfers a PenToken to the address of the binary object or the segment to decrease the reputation score. Each event token also contains the tuple of: (1) the fingerprint of the observed O; (2) the delivery time; and (3) the delivery segment ID. Depending on the cause of violations, a watchdog sends PenToken to the address of binary objects or segments. When a delayed forward and route attack is the cause, the watchdog sends PenToken to the address of the segment. As for the packet modification, the watchdog sends PenToken to both the segment and the binary object. The judge can mark the binary object as corrupted if the invariant part (code and specification) is modified. The delivery time is doubly secured by the block creation time of the transaction for the token transfer since the block creation time can be used as a secure timestamp for the occurrence of the transaction.

### 4.4. Recipient

When a binary object O arrives at the final destination, the recipient first queries the validity of the binary object to a judge. The judge then replies to it by sending the context of the binary object, C(O). Once the binary object is decided as invalid (i.e., non-positive reputation score), the recipient cannot get a legitimate context for the binary object. Since the size of the context is much smaller than the binary object, we assume that the recipient can retrieve the context independently and the recipient can authenticate the response from the judge on blockchain. Before applying the received binary objects, the recipient finally checks the following conditions with the context:

1. Is the identity binding valid? The recipient checks whether the embedded address of the received binary object O and the address of the context are the same. If the binary object is encrypted, the recipient first decrypts it to get the binary in plain text.
2. Is the integrity of the binary preserved? The recipient matches the fingerprint of the received binary object and that in the context.
3. Does the specification in the context show that the binary object is applicable to the recipient’s system? The specification may have an expiration time.

### 4.5. Judge

The judge is the final evaluator regarding both binary objects and segments. It is implemented as smart contracts on the blockchain and triggered when the balance of the participants is updated. To distinguish unauthorized actions, the judge manages the following tasks:

**Context building.** The judge stores the initial context of the binary objects in the blockchain and updates the context from the received information via transactions. It also controls the token system to manage the event tokens from watchdogs and recipient nodes. The transfer and context records stored in the blockchain forms a verifiable trace of the binary objects.

**Reputation Score.** As mentioned in Section 3.2, the reputation score is defined for binary objects and segments. If the reputation score goes below zero (negative), the judge marks it as invalid. An invalid binary object cannot be used since the judge refuses to provide its context. On identifying a corrupted binary object, a watchdog transfers PenToken to the address of the binary object or segment indicated in the token if the segment is responsible for the corruption. Once the reputation score of the segment is less than zero, it is considered to be compromised and then informed through the blockchain system. (For example, Ethereum provides Events and Logs methods for this purpose.) Further actions on the malicious node would be dependent on the service providers and the network operators, such as isolating the corresponding routes.

**Integrity check.** The judge performs a network-wide integrity check with the fingerprint in the context. Due to the nature of in-network monitoring, it is hard for distributed watchdogs to cover a whole area without any shadows. The judge can help to find unnoticeable modification once the fingerprint is registered by benign watchdogs.

**Evaluator review.** Another important role of the judge is to check whether an evaluator improperly reports events. The misbehavior of an evaluator can also be modeled as that of a node. The judge checks that, if an evaluator reports twice as often as the average of other evaluators, we reduce the token issuing rate of the evaluator by half. The issuing rate gradually increases if the evaluator does not preform the suspicious reports anymore.

## 5. IndWatch Model

We describe our design in the TLA^+^ [39] specification language. In the distributed systems, the specification using TLA^+^ is advantageous for checking safety conditions of the distributed systems and algorithms [40,41], and it is also effective for blockchain protocols and applications. Thus, TLA^+^ is being actively used in academia [42,43,44] and industry [45,46].

We express the specification of IndWatch in Temporal Logic, which is used to specify the behaviors of the system in TLA^+^ [47]. We simplified the specification by replacing a complicated formula with a text description (e.g., Next state is $N_{N}ormalSend$). In this way, we can more exactly describe the model itself without losing the readability because of expressing the model at the simpler first-order logic level. Readers are referred to Appendix A for the whole specification of IndWatch. (Detailed information on TLA^+^ can be found in [39]. We briefly describe the core part of the specification in this section but understanding the entire specification is helpful to gain a comprehensive grasp.)

A state is a set of assignments for all (state) variables. We then describe the possible state transitions from the initial states. The main state transition is expressed as: $Spec\;\;\overset{\Delta }{=}\;\;Init\;\wedge \;□{\left[Next\right]}_{vars},$ where Init and Next are the formulas for the initial state and the state transitions, respectively. The transitions are described in the formula Next. The transition usually consists of two parts: enabling conditions and actions. The former is the conditions that must be satisfied to go into the step. An action is a formula that shows the state changes; primed and unprimed variables stand for the state before and after, respectively. The logical operations ∧ and ∨, respectively, stand for conjunction (logical AND) and disjunction (logical OR) of the terms. The operator □ is a temporal operator for ‘always true’ and the action operator ${\left[A\right]}_{e}$ indicates that *A* is true or *e* is not changed (i.e., $e^{\prime }=e$). Thus, $□{\left[Next\right]}_{vars}$ means the state transitions described in Next happens or the system stays at the current state.

Next can be represented as a disjunction of the possible next moves. In the following formula, one of the seven possible state transitions can be selected if only the enabling condition is satisfied. The possible actions for an entity of Segment, Receiver, and Watchdog are also described. The action for blockchain and the judge are specified in ConfirmTx. Finally, Termination indicates the termination conditions without any prime variable.

$\;Next\;\;\overset{\Delta }{=}\;\;\;$

  ∨ PacketGen 

  ∨ ∃n  ∈ Segment : SegmentRecv(n) ∨ SegmentNormalSend(n) 

  ∨ SegmentPacketDrop(n) ∨ SegmentModification(n) 

  ∨ SegmentOnOffForwarding(n) 

  ∨ ∃r  ∈ Receiver : ReceiverRecv(r) 

  ∨ ∃w  ∈ Watchdog : WatchdogSeen(w) ∨ WatchdogCheck(w) 

  ∨ (AllProcessed ∧ ∃w  ∈ Watchdog :  

 wBuffer[w] ≠ {} ∧ WatchdogReview(w)) 

  ∨ ConfirmTx 

  ∨ Termination 

### 5.1. Segment

In the model, we abstract each node as a segment. The actions of segments are straightforward. A segment receives a packet if its immediate destination, idst, indicates the segment and stores it in its buffer, nBuffer, at the SegmentRecv step. Then, at the next step of the node, SegmentNormalSend can be triggered. To model random processing times and malicious nodes, the behavior of the segment is separated into two steps (we add malicious node types, such N_PacketDrop, N_Modification, and N_OnOff in Section 6.1 and use them alternatively for SegmentNormalSend). The segment sends the packet to the next segment (or receiver) according to its routing table, RT, which is an external oracle of the model (given as a parameter during the model checking). The operator Send sends the packet to a shared channel, channel.

$\;SegmentRecv(n)\;\;\overset{\Delta }{=}\;\;\;$

  ∧ nState[n] = N_Wait 

  ∧ ∃p  ∈ channel :  

  ∧ p.idst = n 

  ∧ Receive(p) 

  ∧ (Store the packet to its buffer in nBuffer) 

  ∧ (Next state is N_NormalSend) 

  ∧ UNCHANGED 〈(all the other variables)〉 

$\;SegmentNormalSend(n)\;\;\overset{\Delta }{=}\;\;\;$

  ∧ nState[n] = N_NormalSend 

  ∧  LET  

$\;\;\;\;\;p\;\;\overset{\Delta }{=}\;\;(\mathrm{Get}\;a\;\mathrm{packet}\;\mathrm{from}\;\mathrm{its}\;\mathrm{nBuffer})\;$

$\;\;\;\;\;np\;\;\overset{\Delta }{=}\;\;(\mathrm{Update}\;\mathrm{packet}’s\;\mathrm{information})\;$

    IN  Send(np) 

  ∧ (Remove the processed packet from its nBuffer) 

  ∧ (Next state is N_Wait) 

  ∧ UNCHANGED 〈(all the other variables)〉 

### 5.2. Evaluators

The specification for evaluators has two common parts, First, it monitors the behaviors of segments. Second, when an event occurs, it generates a transaction to transfer a token. Since IndWatch adds the protection by overlaid entities, we give most features to the watchdog and blockchain parts and add a simple function only for the recipient.

A recipient receives a packet at ReceiverRecv and stores it to the buffer in rBuffer. Then, it sends a RepoToken to the address of the received packet (binary object) with the mark of the packet’s fingerprint.

$\;ReceiverRecv(r)\;\;\overset{\Delta }{=}\;\;\;$

  ∧ rState[r] = R_Wait 

  ∧ ∃p  ∈ channel :  

  ∧ p.idst = r 

  ∧ Receive(p) 

  ∧ (Store the packet to its buffer in rBuffer) 

  ∧ (Transfer Repo Token to the received packet) 

  ∧ UNCHANGED 〈(all the other variables)〉 

Whenever a watchdog finds a packet is in transit within its coverage, it stores the packet in the buffer wBuffer at WatchdogSeen. With the stored packets, it evaluates the behaviors of nearby segment (nodes) at WatchdogCheck, which can be triggered whenever there is a matching pair of packets in wBuffer. ReliableDelivery at WatchdogCheck inspects whether the pair of packets have unchanged payloads and have been transmitted within the predefined threshold, NormalTxTime.

$\;WatchdogSeen(w)\;\;\overset{\Delta }{=}\;\;\;$

 ∃p  ∈ channel :  

  ∧ wState[w] = W_Working 

  ∧ p  ∉ wProcessed[w] 

  ∧ (Packet transmission is within the communication range) 

  ∧ (Store the packet to its buffer wBuffer) 

  ∧ UNCHANGED 〈(all the other variables)〉 

$\;WatchdogCheck(w)\;\;\overset{\Delta }{=}\;\;\;$

 ∃p1  ∈ wBuffer[w] : ∃p2  ∈ wBuffer[w] :  

  ∧ p1 ≠ p2 

  ∧ p1.idst = p2.isrc 

  ∧ ReliableDelivery(p1,p2) 

  ∧ Transfer(w,p1.idst,RToken,1,GetFingerprint(p2)) 

  ∧ (Remove p1 and p2 from wBuffer) 

  ∧ (Add p1 and p2 to wProcessed) 

  ∧ UNCHANGED 〈(all the other variables)〉 

$\;WatchdogReview(w)\;\;\overset{\Delta }{=}\;\;\;$

 ∃p  ∈ wBuffer[w] :  

  ∧ IFp.idst  ∈ Node 

      THEN Transfer(w,p.idst,PToken,1,GetFingerprint(p)) 

      ELSE UNCHANGED txPool 

  ∧ (Remove the packet p from wBuffer) 

  ∧ (Add p to wProcessed) 

  ∧ UNCHANGED 〈(all the other variables)〉 

When nodes in a segment relay a packet, the watchdog can see a pair of incoming and outgoing transmissions. If a watchdog monitors any incomplete pairs for a packet, it can be a clue for packet drops or fabrication. Thus, a watchdog regularly performs WatchdogReview, where it marks it as a misbehavior and sends a PenToken to the node. However, for simplicity of the model, we assume that the review process is performed whenever all packets are processed (AllProcessed in Next).

### 5.3. Blockchain System (Token and Judge)

The token system on the blockchain is also an independent entity and its actions are triggered whenever the enabling conditions are satisfied. The main action in ConfirmTx is to validate incoming transactions. For every incoming transaction in txPool, the miners (verifier) of the blockchain check whether the transaction is valid. Since every miner has the same criteria for the valid transaction, we can remove the transaction from txPool once it is processed regardless of its validity. If the transaction is valid, it is moved to txBlock. The token system then applies the change to itself and triggers the decision process of J described in Section 4.5.

$\;ConfirmTx\;\;\overset{\Delta }{=}\;\;\;$

 ∃tx  ∈ txPool :  

$\;\;\;\wedge \;txPool{\;}^{\prime }\;\;=\;txPool\;\;\backslash \;\;\left\{tx\right\}\;$

  ∧ IFCheckBalance(tx.from,tx.token,tx.value) THEN  

$\;\;\;\mathrm{LET}\;e\;\;\overset{\Delta }{=}\;\;tx.from\;n\;\;\overset{\Delta }{=}\;\;tx.to\mathrm{IN}\;\;\;\;$

$\;\;\;\wedge \;txBlock{\;}^{\prime }\;\;=\;txBlock\;\cup \;\left\{tx\right\}\;$

  ∧ (Move tx.token from tx.from to tx.to) 

  ∧ (Register the fingerprint of a newly observed packets to fPrint) 

  ∧ IFn  ∈ Segment ∪ PacketObject ⇒  ∧ JudgeDecision(n) 

  ∧ IntegrityCheck(tx.fingerprint) 

     THEN DecideBenign(n) 

     ELSE  ∧ DecideMalicious(n) 

     ∧ (Mark the packet malicious) 

  ∧ e  ∈ Evaluator 

   ∧ balance[e][tx.token] ≤ TokenMin 

   ⇒ EvaluatorReview(e,tx.token) 

   ELSE UNCHANGED 〈txBlock,balance〉 

  ∧ UNCHANGED 〈(all the other variables)〉 

A valid transaction changes the balance of the evaluator (tx.from) and the segment or the binary object (tx.to) and updates the information for decisions and integrity checks (fPrint). Based on the information, J decides whether the segment or the packet is malicious or not. JudgeDecision checks whether the calculated reputation score is below zero. IntegrityCheck checks whether the fingerprint of the packet is the same as that in the registered fingerprint in fPrint.

## 6. Evaluation

In the evaluation, we aim to answer the following research questions:

- Is this system correctly designed for distributed environments (Section 6.1)?
- Can this system securely deliver software to IIoT devices (Section 6.2)?
- Is this system efficient (Section 6.3)?

For the first question, we use model checking to evaluate the correctness of the design in Section 4. The latter two questions can be answered by experiments: we performed simulations that were implemented on top of the Ethereum system.

### 6.1. Correctness of the Design

The correctness is critically evaluated for the distributed system. It can be evaluated in two aspects: liveness and safety. Liveness guarantees that the desired features will eventually happen. Otherwise, safety guarantees that the system will not go into the erroneous (or bad) states. To check the eventual behaviors, we use model checking. The model checker traverses all possible states of the given model. Thus, we can verify that the system will reach the desired state and will not violate the predefined security conditions. We already employed the model checker while developing the specification in Section 4 and removed dead lock cases.

**Liveness.** Eventually, all software updates should be delivered to their destination if there is no attack. SuccessfullyDelivered indicates the liveness condition and checks whether all outstanding packets arrive at destination without any modification as follows:

$\;SuccessfullyDelivered\;\;\overset{\Delta }{=}\;\;\;$

  ∧ ∀r  ∈ Receiver :  

    ∀p  ∈ {rBuffer[r][x] : x  ∈ DOMAINrBuffer[r]} :  

    checksum[p.cksum] = 〈r,p.data〉 

  ∧ ∀sentPacket  ∈ PreGenPackets \ pendingPackets :  

    ∃r  ∈ Receiver :  

    sentPacket.payload  ∈  

    {rBuffer[r][x].data : x  ∈ DOMAINrBuffer[r]} 

In the presence of attacks, the system filters out illegitimate updates. To successfully identify them, the packet should be registered to the judges properly via benign watchdogs. We set a property, WorkingProperly, to define the condition where all transmitted packets (in sentPackets) are delivered or classified as malicious packets (in malPackets). The condition that a packet is registered by benign watchdogs is represented as IsPacketSeenFirst and the delivery of the packets is checked by IsSecurelyDelivered.

$\;IsPacketSeenFirst(p)\;\;\overset{\Delta }{=}\;\;\;$

  ∧ ∃w  ∈ Watchdog : p  ∈ wBuffer[w] 

  ∧ ∀n  ∈ Node : p  ∉ {nBuffer[n][np] : np  ∈ DOMAINnBuffer[n]} 

$\;IsSecurelyDelivered(p)\;\;\overset{\Delta }{=}\;\;\;$

 ∃r  ∈ Receiver : 〈p.id,p.data〉  ∈ {(Receiver Buffer)} 

$\;WorkingProperly\;\;\overset{\Delta }{=}\;\;\;$

 ∀p  ∈ sentPackets :  

 IsPacketSeenFirst(p) ↝  ∨ p.id  ∈ malPackets 

  ∨ IsSecurelyDelivered(p) 

**Safety.** First, we check whether the unintended evaluation is done by other than the judge entities. The erroneous condition is represented as tokens minted or burned by unauthorized attackers. It causes the changes of total balance for all participants. Thus, we set an invariant property, which must always be true, to see that the total balance is preserved. It is referred to as TokenBalance and defined as follows:

$\;TokenBalance\;\;\overset{\Delta }{=}\;\;\;$

$\;\;\;\mathrm{LET}\;totalBalance\;\;\overset{\Delta }{=}\;\;InitialBalance\;*\;Cardinality(Participant)\;$

  IN Sum(balance,DOMAINbalance) = totalBalance 

We use the TLC model checker for TLA^+^ for the liveness and safety properties. To setup attacks for model checking, we define four node types: N_NormalSend for a benign node, N_PacketDrop for a packet-dropping node, N_Modification for a payload-modifying node, and N_OnOff) for an (on–off) selective-forwarding node. We repeated model checking by adding node of different types.

As a result, the TLC model checker visited 5,100,345 distinct states from our model. We could verify that all the liveness and safety properties are satisfied. In other words, we could confirm that the specification of IndWatch works correctly for the given properties.

### 6.2. Security Analysis

#### 6.2.1. Setup

We evaluated IndWatch against possible attacks with experiments. We simulated the IIoT network with the implementation of the token system and the judge in the smart contract. The judge operation is triggered when the token balance is changed (i.e., on token transfer events by watchdogs.) If the judge finds a malicious node, it generates an Ethereum event to notify the malicious node publicly. The IIoT nodes were simulated in Python. The watchdogs interact with blockchain entities by using the web3py module. The Ethereum blockchain part uses the geth (v1.9.25) client and we set up a two-node private Ethereum network with nodes of Intel i9-9900K with 32 GB Ram. As for the block producing setting, we used PoA (Proof of Authority) rather than PoW (Proof of Work) to control the experiment environments more easily (which was used in the experiments on performance). The IIoT network was simulated by randomly distributing nodes and watchdogs in a field of 1000 m × 1000 m area. The provider is located at the center of the field while the receivers are located randomly in the area. The behavior of each node is implemented based on the model in Section 5.

#### 6.2.2. Attack Models

In simulation, we consider the following attack models for a software supply chain by compromised nodes:

1. **Payload modification** is a traditional attack that modifies the contents of the binary objects. We model it as flipping random bits of the contents.
2. **Target mismatching** is an attack that delivers legitimate binary object to wrong target devices in order to cause malfunction of the victims. We model this as replacing a receiver (each receiver is a unique target in the simulation).
3. **Out of order delivery** is similar to the target mismatching attack: the attacker sends a legitimate binary object to a matched target but swaps it with old one. The old software updates may contain disclosed vulnerabilities that the attacker will use. We model this as replacing the binary object with the previously delivered binary object.
4. **Delayed delivery** is an attack that delays software update deliberately to increase attack time windows for the old vulnerabilities. We model it as delaying for pre-defined time (which is effectively similar to packet dropping due to the timeout of watchdogs).

In distributed monitoring, a watchdog is another attack surface. Physical attacks such as node capture attacks to extract private keys from the watchdog devices, can be prevented by tamper-proof hardware. However, we consider that unauthorized watchdogs can be added to the IIoT network. The previous CIDS approaches with majority voting should be vulnerable to the attacks from malicious (or compromised) watchdogs if they do not have proper authentication mechanisms. Since the blockchain in IndWatch authenticates messages from all entities, unauthorized reports from malicious watchdogs are filtered out. The influence by malicious watchdogs is analyzed in Section 6.2.4. Additionally, we could assume that a judge is not compromised. The core logic of the judge is implemented as a smart contract and operates on the blockchain. Thus, the behavior of judge is publicly verifiable and cannot be altered.

#### 6.2.3. Attack Detection Rate

**Figure 2:**
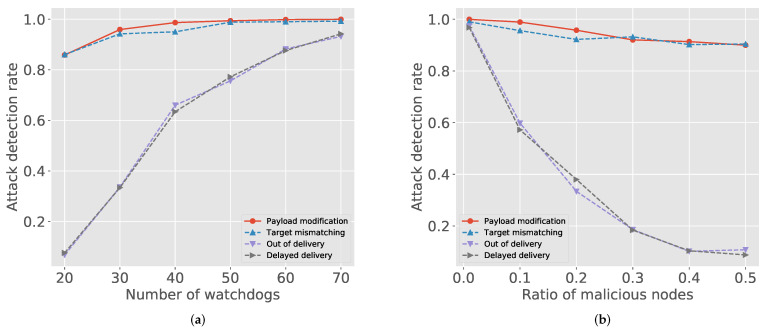
Attack and malicious node detection rate: (**a**) number of watchdogs vs. attack detection rate; and (**b**) number of malicious nodes vs. attack detection rate.

We implemented the attack model regarding malicious nodes for our simulation. For 500 randomly selected sessions, Figure 2a depicts the attack detection rate with a varying number of watchdogs when the number of deployed nodes is 100. The payload modification attack has the highest detection rate because it can be detected by the receiver and the intermediate nodes at the same time. In IndWatch, the blockchain is used for both registration and final decision—unauthorized modifications are easily detected. Thus, the target mismatching is also eventually identified if only the update package is properly registered. However, the delivery attacks (i.e., out-of-delivery and delayed delivery) are mostly detected by the in-network watchdogs, thus their attack detection rates are more sensitive to the change of watchdogs. Thus, we can classify the attack models into two classes by the dependency on watchdogs: Packet modification and out-of-order delivery are representative models for each category that we use in the later results.

We can find similar trends in Figure 2b. The first two attacks, packet modification and target mismatching, are well detected (higher than 0.9) regardless of the number of malicious nodes. However, the detection rate of the latter two attacks, out-of-order delivery and delayed delivery, rapidly decreases as the ratio of malicious nodes increases. Thus, depending on attack type, keeping a certain level of watchdogs is important in IndWatch.

**Figure 3:**
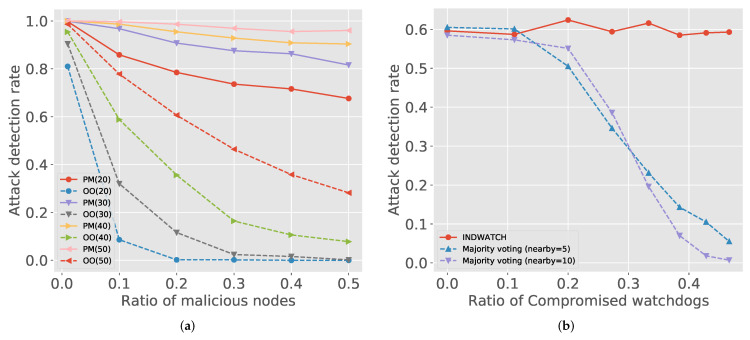
Comparisons of attack detection rates. (**a**) Attack detection rate with different watchdog setting. PM and OO indicate packet modification and out-of-order delivery, respectively. The number in parenthesis is the number of watchdogs. (**b**) Comparison to other distributed watchdogs approaches.

Figure 3a shows the influence of watchdogs and malicious nodes together. We checked the two representative attack models by varying the number of watchdogs and malicious nodes. Figure 3a shows that, even in the hostile environments, where the ratio of malicious nodes is high, increasing the number of watchdogs is effective for both attack classes.

#### 6.2.4. Comparison to CIDS

The blockchain is widely applied to IIoT but an in-network protection mechanism, such as a watchdog, still relies on the distributed approaches adopted from wireless sensor networks [48]. The distributed approaches, referred to as CIDS [18,19], are based on the majority voting of local watchdogs. When a malicious action is detected by multiple nearby watchdogs, the majority of the nearby voting members (i.e., watchdogs) have to confirm that the action is malicious.

Figure 3b compares the attack detection rate between IndWatch and the majority-voting approaches. Malicious watchdogs disturb the consensus of majority voting to reach valid agreements. In Figure 3b, as the portion of malicious watchdogs increases, the attack detection rate is rapidly going down for the majority voting scheme. The decrement is more severe when quorum is high since the decision is influenced by more nodes. IndWatch can authenticate the malicious watchdogs by checking digital signatures in transactions, and thus it can keep a high detection rate regardless of malicious watchdogs.

### 6.3. Performance

Blockchain enables message authenticity and public verifiability but it has a limitation regarding its performance overhead. Transactions can be applied to the system only when it enters into a block, which is created regularly in most of the blockchain [29]. The interval between block creations is called “block time”. The block time in Ethereum is in the order of magnitude of seconds. Furthermore, since the size of a single block is limited (or in terms of execution fee, i.e., gas), the throughput is also limited. Thus, the blockchain may cause dominant latency of the whole process.

We measured the performance of IndWatch through our experiments with local blockchain. Publicly open (i.e., permissionless) blockchain systems are capacity-limited and not suitable for intrusion detection for industrial applications. Thus, we built a local Ethereum blockchain with parameter tweaks to improve the base performance for industrial settings, achieving more than 950 transactions per second.

**Figure 4:**
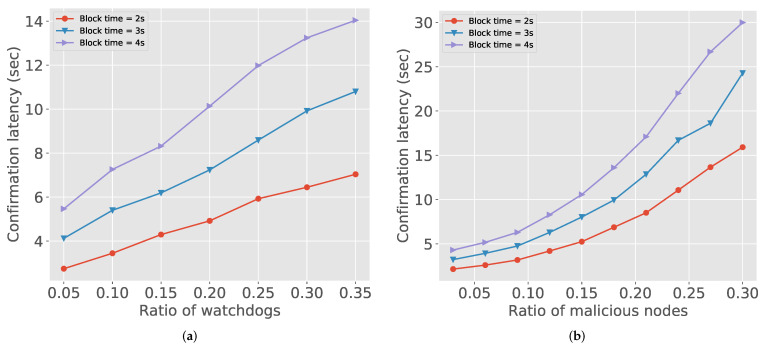
Confirmation latency: (**a**) number of watchdogs vs. confirmation latency; and (**b**) number of malicious nodes vs. confirmation latency.

In Figure 4, we check the confirmation latency with different blockchain settings. The confirmation latency is the end-to-end delay from when packet delivery starts to when the receiver concludes that the delivered packet is legitimate. We use the PoA (Proof-of-Authority) block for blockchain since it is widely used in the enterprise blockchain setup and eligible to control the experiment settings. Figure 4a depicts that, as the portion of watchdogs increases, the confirmation latency increases. This is because more watchdogs generate more reports. When the block time increase, the latency also rapidly increases. The transactions accumulated by watchdogs take longer time to process in this restriction of blocks. Figure 4b also shows the similar trends. As the number of malicious nodes is getting higher, watchdogs could observe more malicious actions. However, compared to Figure 4a, the latency is even higher than 20 s. Therefore, considering the limitations of the current blockchain implementations, blockchain based approaches such as IndWatch should carefully choose application settings, e.g., target detection rate and latency.

## 7. Conclusions and Future Work

Since industrial environments lack decent protection for OTA software updates, in-network protection is practical and applicable to legacy industrial systems. In this study, we proposed a decentralized in-network protection method, IndWatch, by employing the blockchain system to authenticate reports and improve malicious node detection. Through model checking and a simulation based on the existing system, we checked the correctness and operability of IndWatch. We expect that advances in IIoT will increase the demands for secure OTA updates and network protection.

## Appendix A

This appendix contains the whole formal specification of IndWatch. Readers are referred to Section 5 for explanations.

 CONSTANTProvider 

 CONSTANTSegment 

 CONSTANTMiner 

 CONSTANTReceiver 

 CONSTANTJudge 

 CONSTANTWatchdog 

 CONSTANTPreGenPackets 

 CONSTANTPacketObject 

 CONSTANTRToken,PToken 

 CONSTANTInitialBalance 

 CONSTANTRegenTokenMin 

 CONSTANTTokenMin 

 CONSTANTN_Wait,N_NormalSend 

 CONSTANTN_PacketDrop,N_Modification,N_OnOff 

 CONSTANTR_Wait,R_Eval,R_Done 

 CONSTANTW_Working 

 CONSTANTRT 

 CONSTANTCoverage 

 CONSTANTPacketMod 

 VARIABLE nState,rState,wState 

 VARIABLE nBuffer,rBuffer,wBuffer 

 VARIABLE wProcessed 

 VARIABLE channel 

 VARIABLE txPool,txBlock 

 VARIABLE pendingPackets,sentPackets 

 VARIABLE checksum,checksumId 

 VARIABLE pId 

 VARIABLE balance 

 VARIABLE malicious,benign,malPackets 

 VARIABLE fPrint 

 VARIABLE firstSeen 

$\;genVars\;\;\overset{\Delta }{=}\;\;\langle pendingPackets,\;sentPackets,\;pId,\;checksum,\;checksumId\rangle \;$

$\;commVars\;\;\overset{\Delta }{=}\;\;\langle channel\rangle \;$

$\;nodeVars\;\;\;\overset{\Delta }{=}\;\;\langle nState,\;nBuffer\rangle \;$

$\;receiverVars\;\;\;\overset{\Delta }{=}\;\;\langle rState,\;rBuffer\rangle \;$

$\;watchdogVars\;\;\overset{\Delta }{=}\;\;\langle wState,\;wBuffer,\;wProcessed,\;firstSeen\rangle \;$

$\;blockchainVars\;\;\overset{\Delta }{=}\;\;\langle txPool,\;txBlock,\;balance,\;malicious,\;benign,\;\;$

 fPrint,malPackets〉 

$\;vars\;\;\overset{\Delta }{=}\;\;\langle genVars,\;commVars,\;nodeVars,\;receiverVars,\;watchdogVars,\;\;$

 blockchainVars〉 

 RECURSIVE Sum(_,_) 

$\;Sum(f,\;S)\;\;\overset{\Delta }{=}\;\;\mathrm{IF}S\;=\;\{\}\;\mathrm{THEN}\;0\;$

$\;\;\;\;\mathrm{ELSE}\;\;\mathrm{LET}\;x\;\;\overset{\Delta }{=}\;\;\mathrm{CHOOSE}\;x\;\;\in \;S\;:\;\mathrm{TRUE}\;$

   IN f[x] + Sum(f,S \ {x}) 

$\;Send\left(p\right)\;\;\overset{\Delta }{=}\;\;channel{\;}^{\prime }\;\;=\;channel\;\cup \;\left\{p\right\}\;$

$\;Receive\left(p\right)\;\;\overset{\Delta }{=}\;\;channel{\;}^{\prime }\;\;=\;channel\;\;\backslash \;\;\left\{p\right\}\;$

$\;Transfer(src,\;dst,\;ty,\;amount,\;f)\;\;\overset{\Delta }{=}\;\;\;$

$\;\;\;\mathrm{LET}\;\;tx\;\;\overset{\Delta }{=}\;\;[from\;\mapsto \;src,\;to\;\mapsto \;dst,\;\;$

 token ↦ ty,value ↦ amount,fingerprint ↦ f] 

$\;\;\;\;\mathrm{IN}\;txPool{\;}^{\prime }\;\;=\;txPool\;\cup \;\left\{tx\right\}\;$

$\;CheckBalance(address,\;ty,\;amount)\;\;\overset{\Delta }{=}\;\;\;$

 balance[address][ty] ≥ amount 

$\;Evaluator\;\;\overset{\Delta }{=}\;\;Receiver\;\cup \;Watchdog\;$

$\;Participant\;\;\overset{\Delta }{=}\;\;Segment\;\cup \;Evaluator\;\cup \;PacketObject\;$

$\;TokenType\;\;\overset{\Delta }{=}\;\;\{RToken,\;PToken\}\;$

$\;NormalTxTime\;\;\overset{\Delta }{=}\;\;1..10\;$

$\;GetFingerprint(p)\;\;\overset{\Delta }{=}\;\;\langle p.id,\;p.dst,\;p.data,\;p.idst\rangle \;$

$\;InitPacketGen\;\;\overset{\Delta }{=}\;\;\;$

  ∧ checksumId = 1 

  ∧ checksum = [i  ∈ checksumId..   

    (checksumId + Cardinality(PreGenPackets)) ↦ 〈〉] 

  ∧ pId = PacketObject 

  ∧ pendingPackets = PreGenPackets 

  ∧ sentPackets = {} 

$\;InitSegment\;\;\overset{\Delta }{=}\;\;\;$

  ∧ nState = [n  ∈ Segment ↦ N_Wait] 

  ∧ nBuffer = [n  ∈ Segment ↦ 〈〉] 

$\;InitReceiver\;\;\overset{\Delta }{=}\;\;\;$

  ∧ rState = [r  ∈ Receiver ↦ R_Wait] 

  ∧ rBuffer = [r  ∈ Receiver ↦ 〈〉] 

$\;InitWatchdog\;\;\overset{\Delta }{=}\;\;\;$

  ∧ wState = [w  ∈ Watchdog ↦ W_Working] 

  ∧ wBuffer = [w  ∈ Watchdog ↦ {}] 

  ∧ wProcessed = [w  ∈ Watchdog ↦ {}] 

  ∧ firstSeen = {} 

$\;InitBlockchain\;\;\overset{\Delta }{=}\;\;\;$

  ∧ txPool = {} 

  ∧ txBlock = {} 

  ∧ malicious = {} 

  ∧ benign = Participant 

  ∧ balance =  

    [e  ∈ Participant ↦ [ty  ∈ TokenType ↦ InitialBalance]] 

  ∧ fPrint = [id  ∈ PacketObject,p  ∈ Participant ↦ {}] 

  ∧ malPackets = {} 

$\;Init\;\;\overset{\Delta }{=}\;\;\;$

  ∧ InitPacketGen 

  ∧ InitSegment 

  ∧ InitReceiver 

  ∧ InitWatchdog 

  ∧ InitBlockchain 

  ∧ channel = {} 

$\;PacketGen\;\;\overset{\Delta }{=}\;\;\;$

 ∃p  ∈ pendingPackets : ∃n  ∈ Segment : ∃pp  ∈ pId :  

$\;\;\;\mathrm{LET}\;msg\;\;\overset{\Delta }{=}\;\;[\;$

 id ↦ pp, 

 isrc ↦ Provider, 

 idst ↦ n, 

 dst ↦ p.dst, 

 data ↦ p.payload, 

 elapsed ↦ 0, 

 cksum ↦ checksumId] 

   IN  

$\;\;\;\wedge \;pendingPackets{\;}^{\prime }\;\;=\;pendingPackets\;\;\backslash \;\;\left\{p\right\}\;$

$\;\;\;\wedge \;pId{\;}^{\prime }\;\;=\;pId\;\;\backslash \;\;\left\{pp\right\}\;$

  ∧ Send(msg) 

$\;\;\;\wedge \;sentPackets{\;}^{\prime }\;\;=\;sentPackets\;\cup \;\left\{msg\right\}\;$

$\;\;\;\wedge \;checksum{\;}^{\prime }\;\;=\;\;$

    [checksum EXCEPT![checksumId] = 〈p.dst,p.payload〉] 

$\;\;\;\wedge \;checksumId{\;}^{\prime }\;\;=\;checksumId\;+\;1\;$

  ∧ UNCHANGED 〈nodeVars,receiverVars,watchdogVars, 

    blockchainVars〉 

$\;SegmentRecv(n)\;\;\overset{\Delta }{=}\;\;\;$

  ∧ nState[n] = N_Wait 

  ∧ ∃p  ∈ channel :  

  ∧ p.idst = n 

  ∧ Receive(p) 

$\;\;\;\wedge \;nBuffer{\;}^{\prime }\;\;=\;\left[nBuffer\;\mathrm{EXCEPT}\;!\;\left[n\right]\;=\;Append\left(@,\;p\right)\right]\;$

$\;\;\;\wedge \;nState{\;}^{\prime }\;\;=\;\left[nState\;\mathrm{EXCEPT}\;!\;\left[n\right]\;=\;N\_\;NormalSend\right]\;$

  ∧ UNCHANGED 〈genVars,receiverVars,watchdogVars, 

 blockchainVars〉 

$\;SegmentNormalSend(n)\;\;\overset{\Delta }{=}\;\;\;$

  ∧ nState[n] = N_NormalSend 

  ∧  LET  

$\;\;\;\;\;p\;\;\overset{\Delta }{=}\;\;Head(nBuffer[n])\;$

$\;\;\;\;\;et\;\;\;\overset{\Delta }{=}\;\;\mathrm{CHOOSE}\;x\;\;\in \;NormalTxTime\;:\;\mathrm{TRUE}\;$

$\;\;\;\;\;np\;\;\overset{\Delta }{=}\;\;[p\;\mathrm{EXCEPT}\;!\;.isrc\;=\;n,\;\;!\;.idst\;=\;RT[n],\;\;$

 !.elapsed = @ + et] 

   IN Send(np) 

$\;\;\;\wedge \;nBuffer{\;}^{\prime }\;\;=\;\left[nBuffer\;\mathrm{EXCEPT}\;!\;\left[n\right]\;=\;Tail\left(@\right)\right]\;$

$\;\;\;\wedge \;nState{\;}^{\prime }\;\;=\;\left[nState\;\mathrm{EXCEPT}\;!\;\left[n\right]\;=\;N\_\;Wait\right]\;$

  ∧ UNCHANGED 〈genVars,receiverVars,watchdogVars, 

 blockchainVars〉 

$\;SegmentPacketDrop(n)\;\;\overset{\Delta }{=}\;\;\;$

  ∧ nState[n] = N_PacketDrop 

$\;\;\;\wedge \;nBuffer{\;}^{\prime }\;\;=\;\left[nBuffer\;\mathrm{EXCEPT}\;!\;\left[n\right]\;=\;Tail\left(@\right)\right]\;$

$\;\;\;\wedge \;nState{\;}^{\prime }\;\;=\;\left[nState\;\mathrm{EXCEPT}\;!\;\left[n\right]\;=\;N\_\;Wait\right]\;$

  ∧ UNCHANGED 〈genVars,receiverVars,watchdogVars,commVars, 

 blockchainVars〉 

$\;SegmentOnOffForwarding(n)\;\;\overset{\Delta }{=}\;\;\;$

  ∧ nState[n] = N_OnOff 

$\;\;\;\wedge \;\;\mathrm{LET}\;b\;\;\overset{\Delta }{=}\;\;\mathrm{CHOOSE}\;x\;\;\in \;\{\mathrm{TRUE},\;\mathrm{FALSEIN}\}\;:\;\mathrm{TRUEIN}\;\;\;\;$

$\;\;\;\;\;nState{\;}^{\prime }\;\;=\;[nState\;\mathrm{EXCEPT}\;!\;\left[n\right]\;$

     = IFb THEN N_NormalSend  ELSE N_PacketDrop] 

  ∧ UNCHANGED 〈genVars,receiverVars,watchdogVars,commVars, 

 nBuffer,blockchainVars〉 

$\;SegmentModification(n)\;\;\overset{\Delta }{=}\;\;\;$

  ∧ nState[n] = N_Modification 

  ∧  LET  

$\;\;\;\;\;p\;\;\overset{\Delta }{=}\;\;Head(nBuffer[n])\;$

$\;\;\;\;\;et\;\;\;\overset{\Delta }{=}\;\;\mathrm{CHOOSE}\;x\;\;\in \;NormalTxTime\;:\;\mathrm{TRUE}\;$

$\;\;\;\;\;np\;\;\overset{\Delta }{=}\;\;[p\;\mathrm{EXCEPT}\;!\;.isrc\;=\;n,\;\;!\;.idst\;=\;RT[p.dst],\;\;$

 !.elapsed = @ + et,!.data = @ + PacketMod] 

   IN Send(np) 

$\;\;\;\wedge \;nBuffer{\;}^{\prime }\;\;=\;\left[nBuffer\;\mathrm{EXCEPT}\;!\;\left[n\right]\;=\;Tail\left(@\right)\right]\;$

$\;\;\;\wedge \;nState{\;}^{\prime }\;\;=\;\left[nState\;\mathrm{EXCEPT}\;!\;\left[n\right]\;=\;N\_\;Wait\right]\;$

  ∧ UNCHANGED 〈genVars,receiverVars,watchdogVars, 

 blockchainVars〉 

$\;ReceiverRecv(r)\;\;\overset{\Delta }{=}\;\;\;$

  ∧ rState[r] = R_Wait 

  ∧ ∃p  ∈ channel :  

  ∧ p.idst = r 

  ∧ Receive(p) 

$\;\;\;\wedge \;rBuffer{\;}^{\prime }\;\;=\;\left[rBuffer\;\mathrm{EXCEPT}\;!\;\left[r\right]\;=\;Append\left(@,\;p\right)\right]\;$

  ∧ Transfer(r,p.id,RToken,1,GetFingerprint(p)) 

  ∧ UNCHANGED 〈genVars,nodeVars,rState,watchdogVars,txBlock, 

 balance,malicious,benign,fPrint,malPackets〉 

$\;ReliableDelivery(p1,\;p2)\;\;\overset{\Delta }{=}\;\;\;$

  ∧ p2.elapsed − p1.elapsed  ∈ NormalTxTime 

  ∧ p1.data = p2.data 

$\;IsPacketSeenFirst(p)\;\;\overset{\Delta }{=}\;\;\;$

  ∧ ∃w  ∈ Watchdog : p  ∈ wBuffer[w] 

  ∧ ∀n  ∈ Segment : p  ∉ {nBuffer[n][np] : np  ∈ DOMAINnBuffer[n]} 

$\;WatchdogSeen(w)\;\;\overset{\Delta }{=}\;\;\;$

 ∃p  ∈ channel :  

  ∧ wState[w] = W_Working 

  ∧ p  ∉ wProcessed[w] 

  ∧ (p.isrc  ∈ Coverage[w] ∨ p.idst  ∈ Coverage[w]) 

$\;\;\;\wedge \;wBuffer{\;}^{\prime }\;\;=\;\left[wBuffer\;\mathrm{EXCEPT}\;!\;\left[w\right]\;=\;@\;\cup \;\left\{p\right\}\right]\;$

  ∧ IFIsPacketSeenFirst(p) 

$\;\;\;\mathrm{THEN}\;firstSeen{\;}^{\prime }\;\;=\;firstSeen\;\cup \;\left\{p\right\}\;\;\mathrm{ELSE}\;\mathrm{UNCHANGED}\;firstSeen\;$

  ∧ UNCHANGED 〈genVars,nodeVars,commVars,receiverVars, 

 wState,wProcessed,blockchainVars〉 

$\;WatchdogCheck(w)\;\;\overset{\Delta }{=}\;\;\;$

 ∃p1  ∈ wBuffer[w] : ∃p2  ∈ wBuffer[w] :  

  ∧ p1 ≠ p2 

  ∧ p1.idst = p2.isrc 

  ∧ ReliableDelivery(p1,p2) 

  ∧ Transfer(w,p1.idst,RToken,1,GetFingerprint(p2)) 

$\;\;\;\wedge \;wBuffer{\;}^{\prime }\;\;=\;\left[wBuffer\;\mathrm{EXCEPT}\;!\;\left[w\right]\;=\;@\;\;\backslash \;\;\left\{p1,\;p2\right\}\right]\;$

$\;\;\;\wedge \;wProcessed{\;}^{\prime }\;\;=\;\left[wProcessed\;\mathrm{EXCEPT}\;!\;\left[w\right]\;=\;@\;\cup \;\left\{p1,\;p2\right\}\right]\;$

  ∧ UNCHANGED 〈genVars,nodeVars,commVars,receiverVars, 

 firstSeen,wState,txBlock,balance,malicious,benign, 

 fPrint,malPackets〉 

$\;WatchdogReview(w)\;\;\overset{\Delta }{=}\;\;\;$

 ∃p  ∈ wBuffer[w] :  

  ∧ IFp.idst  ∈ Segment 

  THEN Transfer(w,p.idst,PToken,1,GetFingerprint(p)) 

   ELSE UNCHANGED txPool 

$\;\;\;\wedge \;wBuffer{\;}^{\prime }\;\;=\;\left[wBuffer\;\mathrm{EXCEPT}\;!\;\left[w\right]\;=\;@\;\;\backslash \;\;\left\{p\right\}\right]\;$

$\;\;\;\wedge \;wProcessed{\;}^{\prime }\;\;=\;\left[wProcessed\;\mathrm{EXCEPT}\;!\;\left[w\right]\;=\;@\;\cup \;\left\{p\right\}\right]\;$

  ∧ UNCHANGED 〈genVars,nodeVars,commVars,receiverVars, 

 firstSeen,wState,txBlock,balance,malicious,benign, 

 fPrint,malPackets〉 

$\;DecideMalicious\left(n\right)\;\;\overset{\Delta }{=}\;\;malicious{\;}^{\prime }\;\;=\;malicious\;\cup \;\left\{n\right\}\;$

$\;DecideBenign\left(n\right)\;\;\overset{\Delta }{=}\;\;benign{\;}^{\prime }\;\;=\;benign\;\cup \;\left\{n\right\}\;$

Return FALSE if the node is malicious

$\;JudgeDecision(n)\;\;\overset{\Delta }{=}\;\;\;$

$\;\;\;\mathrm{LET}\;score\;\;\overset{\Delta }{=}\;\;InitialBalance\;-\;balance[n][PToken]\;$

   IN  

  ∧ (score ≥ 0) 

$\;IntegrityCheck(fp)\;\;\overset{\Delta }{=}\;\;\;$

$\;\;\;\mathrm{LET}\;id\;\;\overset{\Delta }{=}\;\;fp[1]\;dst\;\;\overset{\Delta }{=}\;\;fp[2]\;pfp\;\;\overset{\Delta }{=}\;\;fp[3]\;$

$\;\;storedFp\;\;\overset{\Delta }{=}\;\;fPrint[id,\;dst]\;\;\;\mathrm{IN}\;\;$

 storedFp ≠ {} ⇒ pfp  ∈ storedFp 

 RECURSIVE SumToken(_,_,_) 

$\;SumToken(f,\;S,\;t)\;\;\overset{\Delta }{=}\;\;\;$

 IFS = {} THEN 0  ELSE  

$\;\;\;\mathrm{LET}\;x\;\;\overset{\Delta }{=}\;\;\mathrm{CHOOSE}\;x\;\;\in \;S\;:\;\mathrm{TRUE}\;$

   IN f[x][t] + SumToken(f,S \ {x},t) 

$\;EvaluatorReview(e,\;ty)\;\;\overset{\Delta }{=}\;\;\;$

$\;\;\;\wedge \;\;\mathrm{LET}\;tTotal\;\;\overset{\Delta }{=}\;\;SumToken(balance,\;Evaluator,\;ty)\;\;\mathrm{IN}\;\;$

 IF(2 ∗ balance[e][ty] ∗ Cardinality(Evaluator) < tTotal) 

$\;\;\;\mathrm{THEN}\;balance{\;}^{\prime }\;\;=\;[balance\;\mathrm{EXCEPT}\;$

 ![e] = [@ EXCEPT![ty] = @ + RegenTokenMin]] 

$\;\;\;\;\mathrm{ELSE}\;balance{\;}^{\prime }\;\;=\;[balance\;\mathrm{EXCEPT}\;$

 ![e] = [@ EXCEPT![ty] = @ + 2 ∗ RegenTokenMin]] 

$\;ConfirmTx\;\;\overset{\Delta }{=}\;\;\;$

 ∃tx  ∈ txPool :  

$\;\;\;\wedge \;txPool{\;}^{\prime }\;\;=\;txPool\;\;\backslash \;\;\left\{tx\right\}\;$

  ∧ IFCheckBalance(tx.from,tx.token,tx.value) THEN  

$\;\;\;\mathrm{LET}\;e\;\;\overset{\Delta }{=}\;\;tx.from\;n\;\;\overset{\Delta }{=}\;\;tx.to\;\;\mathrm{IN}\;\;$

$\;\;\;\wedge \;txBlock{\;}^{\prime }\;\;=\;txBlock\;\cup \;\left\{tx\right\}\;$

$\;\;\;\wedge \;balance{\;}^{\prime }\;\;\;=\;[balance\;\mathrm{EXCEPT}\;$

 ![e] = [@ EXCEPT![tx.token] = @ − tx.value], 

 ![n] = [@ EXCEPT![tx.token] = @ + tx.value]] 

$\;\;\;\wedge \;\;\mathrm{LET}\;id\;\;\overset{\Delta }{=}\;\;tx.fingerprint[1]\;dst\;\;\overset{\Delta }{=}\;\;tx.fingerprint[2]\;$

$\;\;sfp\;\;\overset{\Delta }{=}\;\;tx.fingerprint[3]\;\;\mathrm{IN}\;\;$

 IFfPrint[id,dst] = {} 

$\;\;\;\mathrm{THEN}\;fPrint{\;}^{\prime }\;\;=\;\left[fPrint\;\mathrm{EXCEPT}\;!\;\left[id,\;dst\right]\;=\;\left\{sfp\right\}\right]\;$

   ELSE UNCHANGED fPrint 

  ∧ IFn  ∈ Segment ∪ PacketObject ⇒  ∧ JudgeDecision(n) 

  ∧ IntegrityCheck(tx.fingerprint) 

  THEN DecideBenign(n) ∧ UNCHANGED 〈malicious,malPackets〉 

   ELSE  ∧ DecideMalicious(n) 

$\;\;\;\wedge \;malPackets{\;}^{\prime }\;\;=\;malPackets\;\cup \;\left\{tx.fingerprint\left[1\right]\right\}\;$

  ∧ UNCHANGED benign 

  ∧ e  ∈ Evaluator 

  ∧ balance[e][tx.token] ≤ TokenMin 

  ⇒ EvaluatorReview(e,tx.token) 

   ELSE UNCHANGED 〈txBlock,balance〉 

  ∧ UNCHANGED 〈genVars,nodeVars,commVars,receiverVars, 

 watchdogVars〉 

$\;SuccesfullyDelivered\;\;\overset{\Delta }{=}\;\;\;$

  ∧ ∀r  ∈ Receiver :  

 ∀p  ∈ {rBuffer[r][x] : x  ∈ DOMAINrBuffer[r]} :  

  ∨ checksum[p.cksum] = 〈r,p.data〉 

  ∧ ∀sentPacket  ∈ PreGenPackets :  

 ∃r  ∈ Receiver :  

 sentPacket.payload  ∈  

 {rBuffer[r][x].data : x  ∈ DOMAINrBuffer[r]} 

$\;AllDelivered\;\;\overset{\Delta }{=}\;\;\;$

  ∧ ∀r  ∈ Receiver :  

 ∀p  ∈ {rBuffer[r][x] : x  ∈ DOMAINrBuffer[r]} :  

  ∨ checksum[p.cksum] = 〈r,p.data〉 

  ∨ checksum[p.cksum] = 〈r,p.data − PacketMod〉 

  ∧ ∀sentPacket  ∈ PreGenPackets :  

 ∃r  ∈ Receiver :  

 sentPacket.payload  ∈  

 {rBuffer[r][x].data : x  ∈ DOMAINrBuffer[r]} ∪  

 {rBuffer[r][x].data − PacketMod : x  ∈ DOMAINrBuffer[r]} 

$\;AllProcessed\;\;\overset{\Delta }{=}\;\;\;$

  ∧ pendingPackets = {} 

  ∧ ∀n  ∈ Segment : nBuffer[n] = 〈〉 

  ∧ channel = {} 

$\;IsSecurelyDelivered(p)\;\;\overset{\Delta }{=}\;\;\;$

 ∃r  ∈ Receiver :  

 〈p.id,p.data〉  ∈  

 {〈rBuffer[r][rp].id,rBuffer[r][rp].data〉 : rp  ∈ DOMAINrBuffer[r]} 

$\;WorkingProperly\;\;\overset{\Delta }{=}\;\;\;$

 ∀p  ∈ sentPackets :  

 IsPacketSeenFirst(p) ↝  ∨ p.id  ∈ malPackets 

  ∨ IsSecurelyDelivered(p) 

$\;Termination\;\;\overset{\Delta }{=}\;\;\;$

  ∧ txPool = {} 

  ∧ ∀w  ∈ Watchdog : wBuffer[w] = {} 

  ∧ AllProcessed 

  ∧ ∀r  ∈ Receiver : rState[r] = R_Wait 

  ∧ ∀p  ∈ firstSeen : p.id  ∈ malPackets ∨ IsSecurelyDelivered(p) 

  ∧ UNCHANGED vars 

$\;TokenBalance\;\;\overset{\Delta }{=}\;\;\;$

$\;\;\;\mathrm{LET}\;totalBalance\;\;\overset{\Delta }{=}\;\;InitialBalance\;*\;Cardinality(Participant)\;$

   IN Sum(balance,DOMAINbalance) = totalBalance 

$\;Delivered\;\;\;\overset{\Delta }{=}\;\;\;$

 ∀p  ∈ sentPackets :  

 ENABLEDIsPacketSeenFirst(p) ⇒ IsSecurelyDelivered(p) 

$\;Next\;\;\overset{\Delta }{=}\;\;\;$

  ∨ PacketGen 

  ∨ ∃n  ∈ Segment : SegmentRecv(n) ∨ SegmentNormalSend(n) 

  ∨ SegmentPacketDrop(n) ∨ SegmentModification(n) ∨ SegmentOnOffForwarding(n) 

  ∨ ∃r  ∈ Receiver : ReceiverRecv(r) 

  ∨ ∃w  ∈ Watchdog : WatchdogSeen(w) ∨ WatchdogCheck(w) 

  ∨ (AllProcessed ∧ ∃w  ∈ Watchdog :  

 wBuffer[w] ≠ {} ∧ WatchdogReview(w)) 

  ∨ ConfirmTx 

  ∨ Termination 

$\;Spec\;\;\overset{\Delta }{=}\;\;Init\;\wedge \;□{\left[Next\right]}_{vars}\;$
